# Resilient Health Care: a systematic review of conceptualisations, study methods and factors that develop resilience

**DOI:** 10.1186/s12913-020-05208-3

**Published:** 2020-04-17

**Authors:** Mais Iflaifel, Rosemary H. Lim, Kath Ryan, Clare Crowley

**Affiliations:** 1 Reading School of Pharmacy, University of Reading, Reading, Berkshire, UK; 2 Pharmacy Department, Oxford University Hospitals NHS Foundation Trust, Oxford, UK

**Keywords:** Health care, Resilience, Resilient health care, Safety-II, Work as done, Assessment methods, Safety

## Abstract

**Background:**

Traditional approaches to safety management in health care have focused primarily on counting errors and understanding how things go wrong. Resilient Health Care (RHC) provides an alternative complementary perspective of learning from incidents and understanding how, most of the time, work is safe. The aim of this review was to identify how RHC is conceptualised, described and interpreted in the published literature, to describe the methods used to study RHC, and to identify factors that develop RHC.

**Methods:**

Electronic searches of PubMed, Scopus and Cochrane databases were performed to identify relevant peer-reviewed studies, and a hand search undertaken for studies published in books that explained how RHC as a concept has been interpreted, what methods have been used to study it, and what factors have been important to its development. Studies were evaluated independently by two researchers. Data was synthesised using a thematic approach.

**Results:**

Thirty-six studies were included; they shared similar descriptions of RHC which was the ability to adjust its functioning prior to, during, or following events and thereby sustain required operations under both expected and unexpected conditions. Qualitative methods were mainly used to study RHC. Two types of data sources have been used: direct (e.g. focus groups and surveys) and indirect (e.g. observations and simulations) data sources. Most of the tools for studying RHC were developed based on predefined resilient constructs and have been categorised into three categories: performance variability and Work As Done, cornerstone capabilities for resilience, and integration with other safety management paradigms. Tools for studying RHC currently exist but have yet to be fully implemented. Effective team relationships, trade-offs and health care ‘resilience’ training of health care professionals were factors used to develop RHC.

**Conclusions:**

Although there was consistency in the conceptualisation of RHC, methods used to study and the factors used to develop it, several questions remain to be answered before a gold standard strategy for studying RHC can confidently be identified. These include operationalising RHC assessment methods in multi-level and diverse settings and developing, testing and evaluating interventions to address the wider safety implications of RHC amidst organisational and institutional change.

## Background

Globally, it is reported that about 10% of hospitalised patients experience adverse health care events. Health care organisations may struggle to provide safe and high quality care, and as a result, people might experience unintentional harm [1–3].

The traditional approach to increasing safety has focused on counting incidents, identifying system failures, and understanding the causes of incidents in order to develop strategies to eliminate or reduce them [4]. This is called a Safety-I approach [5]. A Safety-II approach, however, recognises that work can be viewed from different perspectives [5]. The closer the viewer is to the work (whether in space, time or knowledge/experience), the more accurate their understanding about how the work is done. As the viewer moves further from the work, their understanding becomes necessarily more simplified and less accurate. This is often conceptualised as a ‘wedge’ with a sharp proximal point and a blunt distal edge. In a health care context, health care professionals interact directly with a hazardous process, representing the actual workplace. Regulators, policy makers and managers control and balance the resources, constraints and multiple demands imposed on health care professionals. Safety problems are not always a direct result of a lack of knowledge or effort by health care professionals − they are usually a result of work that is complex, often involving the use of technology [5]. There is often a mismatch, however, between how everyday work is accomplished (Work As Done) and how work is presumed to have happened (Work As Imagined) [6, 7]. These mismatches might sometimes lead to safety problems and there is, therefore, value in learning from the full range of work outcomes, including usual outcomes (when things go right), negative outcomes (for example, errors) and everything in between, despite the inevitable risks and complexity. This is the core concept of the Safety-II approach [5]. Health care professionals often work under varying conditions using principles of both Safety-I and Safety-II, but policymakers, regulators and/or health care managers typically focus their efforts on standardising work practice based on Safety-I principles. For example, safety efforts often focus on counting and tracking events that fail rather than those that succeed [5, 8].

Resilience engineering (RE) has been advocated since the last decade as a new paradigm for safety management in socio-technical systems [9]. RE focuses on a system’s capacity to cope with complexity and variable conditions [9, 10]. RE has been applied to various disciplines such as aviation, railways, natural disasters, health care and others [11]. Resilient Health Care (RHC) which applies concepts of RE to health care settings and adopts a Safety-II approach, provides a complementary perspective of learning from incidents and understanding how everyday clinical work is successful [12–14]. RHC acknowledges that health care systems such as a clinic, ward, hospital, or even country, are complex adaptive systems that are constantly changing and can result in unexpected work situations. Because they can anticipate, monitor, respond and adapt to threats, health care professionals are viewed as resources and assets rather than as a problem to be solved or standardised. Therefore, the focus is on how everyday clinical work is performed rather than solely on the unpredictable accidents or incidents [4, 5, 12]. RHC does not focus on an individual’s coping and resilience capacity but rather on the factors and methods that enable the workers, team and unit or organisation to adapt and cope effectively in different situations.

RHC is theoretically attractive and recent reviews indicate a growing interest and evidence in operationalising RHC [15, 16] for example in defining models and measurements to understand the effect of trade-offs in operational activities [17]. Ellis et al. (2019) however, reported an increasing shift from studying and understanding RHC to developing resilience in health care settings [18]. There remains, however, conceptual and methodological issues around operationalising RHC. Righi et al. (2015) and Patriarca et al. (2018) highlighted the importance of conceptualising and anchoring resilience to capabilities that characterise resilient systems and explicitly define which capability is under study when describing or modelling resilience, and to develop innovative frameworks that can integrate different existing capabilities to understand in depth their commonalities, differences and relationships [11, 17]. Hollnagel (2006) made a valuable contribution by defining the four capabilities of resilient systems: anticipate, monitor, respond and learn [9]. Other researchers have proposed other resilience capabilities, such as rebound from unexpected events and return to equilibrium, robustness [19], planning, adapting, and noticing [20]. Berg and Aase (2019) conceptualised resilience in healthcare based on four categories: anticipation, sense making, trade-offs and adaptations, and defined it as a set of cognitive and behavioural strategies enacted by individuals within an organisational context [21]. Patriarca et al. (2018) highlighted the importance of developing more advanced safety-oriented models to study resilience in order to overcome the limitations of the traditional safety approaches [17].

Berg et al. (2018) identified methodological issues in the current empirical literature. They found data collection in studies focused primarily on one level, the micro system level for example frontline clinical staff, rather than an integrated understanding of complex, multi-level systems as a whole [22]. They argued for the need to clearly define the resilient construct to develop theoretical frameworks for empirical testing across different system levels. Berg et al. (2018)‘s review was limited to peer-reviewed studies that described the studies’ data collection method because they aimed to synthesise methodological strategies [22]. Various models/methods have been developed and used to study resilience, including modelling activities using fuzzy cognitive maps in petrochemical plants [23], the Benefit−Cost−Deficit model to predict car driving violations [15], and the Functional Resonance Analysis Method (FRAM) to analyse the impact of variability on everyday work [24].

As observed by Ellis et al. (2019), the current literature on RHC has reported factors that promote resilience. Examples include training and educating health care professionals to cope with various conditions [4, 25, 26]; encouraging different departments and specialities to communicate about concerns pertaining to work practice [21, 25, 27]; repeated exposure to similar disturbances [4, 26, 28]; enhancing the knowledge and experience of health care professionals to respond to actual work conditions and to enact important trade-offs [4, 21, 28]; reducing the cognitive load on health care practitioners by simulation training to manage expected and unexpected situations [4]; and integrating human factors and health economics in the design process [16].

Despite recent reviews, there is still no ‘gold standard’ for studying and developing RHC in health care. There is still a lack of understanding of how RHC is conceptualised in empirical studies, for example whether and/or what resilient capabilities are used to conceptualise RHC, the methods/models/frameworks used to study and operationalise RHC, and factors to develop and enhance RHC. It is vital to gather emerging knowledge on applied definitions, methods, models and factors, using a wide range of empirical studies in health care from different sources to provide a robust contribution to the development of RHC research.

As such, the objectives of the systematic review were to: 1. identify how RHC is conceptualised in health care studies; 2. identify and analyse methods and tools used to study RHC; 3. identify and analyse factors that develop and enhance RHC.

## Methods

A protocol for the systematic review was registered with PROSPERO (registration number: PROSPERO 2019 CRD42019129049). This systematic review is reported following the Preferred Reporting Items for Systematic Reviews and Meta-Analysis (PRISMA) guidelines for reporting of systematic reviews [29].

### Ethical approval

Ethical approval was not required as the study was a systematic review of peer-reviewed journal articles and studies published in books.

### Inclusion and exclusion criteria

The systematic review was limited to:

1. Scholarly peer-reviewed journal articles and studies published in books, written in English.
2. Studies published in journals and books that described RHC, and/or methods used to study RHC and/or factors to develop and enhance RHC in any health care setting.

Studies were excluded if:

1. They were about resilience in non-health disciplines.
2. They were about individual or community resilience.
3. They were about resilience in disaster.

### Search strategy and study selection

Electronic searches of PubMed, Scopus, and Cochrane databases were conducted using the following search terms: (organisational/organizational and/or resilien* or safety or safety I or safety 1 or safety II or safety 2 or “work as imagined” or “work as done” and health care or healthcare or hospital) and/or (tool, measure, strateg*, solution). Other search methods such as hand searching, serendipity/browsing, checking with experts, and searching the specialist website resilienthealthcare.net were also used to identify further relevant peer-reviewed studies and studies published in books. The search covered a time period from January 1982 to April 2019. The titles and abstracts of identified studies were screened independently by two researchers (MI and RL) applying inclusion and exclusion criteria specified a priori. Full-text studies of retained references were then obtained and screened independently by three researchers (MI, RL and KR) using the same inclusion/exclusion criteria. Disagreements were resolved by discussion to achieve consensus.

### Data extraction

MI independently extracted the following information: author(s), year of publication, country in which the study was conducted, aim of study, study design and methodology, study setting, sample size, descriptions of RHC, methods used to study RHC and factors that develop RHC where available.

### Quality assessment

The quality of the included studies was evaluated using the Mixed Methods Appraisal Tool (MMAT) version 2018 [30], an established tool that enables critical appraisal of quantitative, qualitative, and mixed methods studies. See Additional file 1 for items assessed. An appraisal of all studies (by MI) and a random selection of a third of the studies (by RL) were conducted. Any disagreements were discussed between MI and RL to reach consensus.

### Data synthesis

Due to the heterogeneity of study designs, it was not possible to use a meta-analysis approach to analyse the quantitative findings. Data was synthesised using both deductive (question 1) and inductive (questions 2 and 3) thematic approach [31]. This entailed the following steps [32]:

1. Familiarisation: studies were read multiple times to ensure familiarity with the content.
2. Developing a coding framework: data were coded line by line based on the key aims of the systematic review, which were descriptions of RHC, methods used to study RHC and factors used to enhance RHC in health care settings.
3. Indexing: shared categories were developed while reading and comparing between different studies.
4. Charting: the coded data and similar findings were grouped into key themes and subthemes within and across studies.
5. Mapping and interpretation: subthemes were aligned to the main theme to provide explanations for the findings.

## Results

### Studies included in the review

**Fig. 1:**
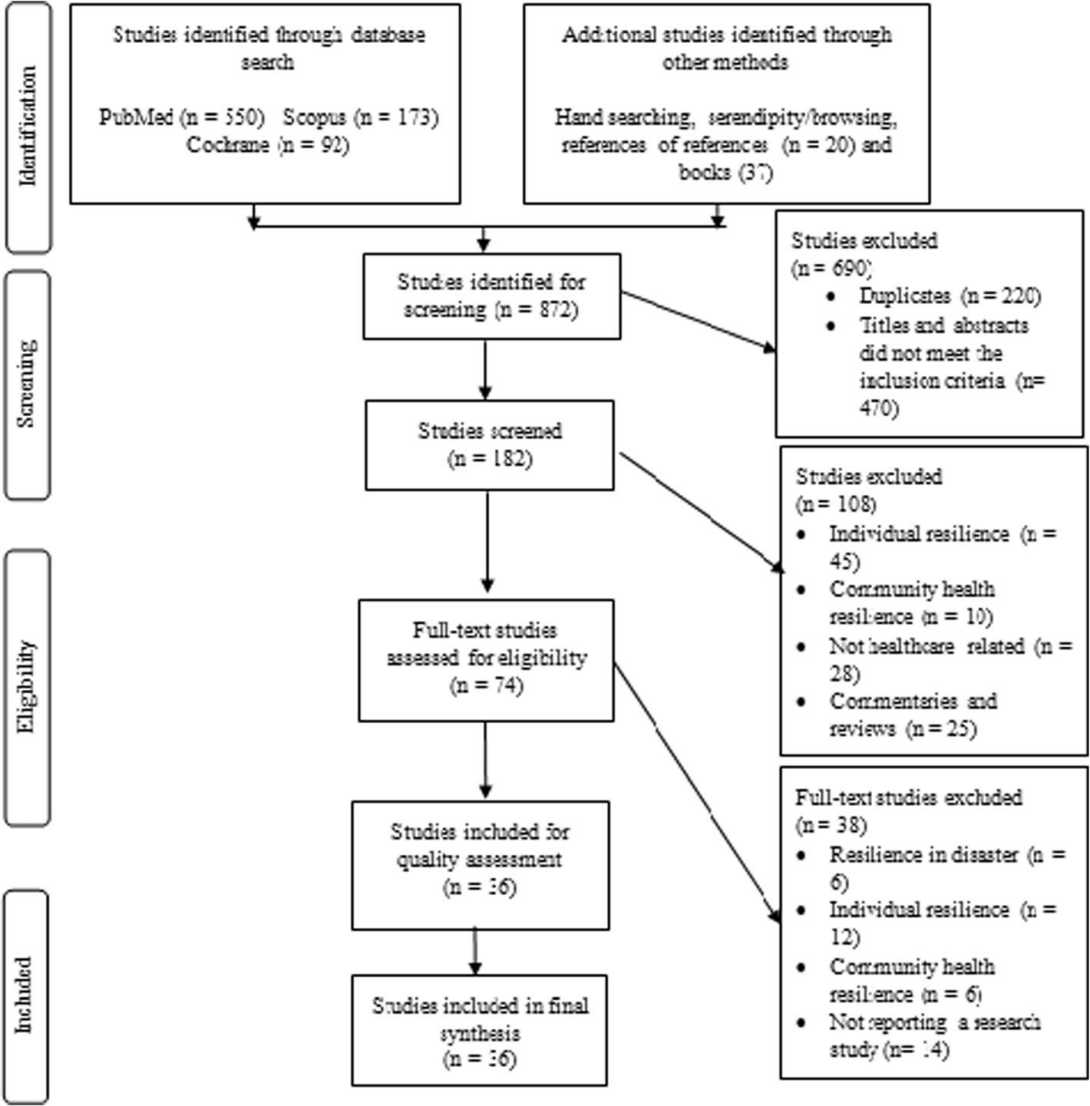
PRISMA flow chart for study selection process

Eight hundred and seventy-two studies were identified in the initial database searches. Following screening, 74 studies were left for full-text review. From these, a total of 20 studies published in peer-reviewed journals and 16 studies published in books were included in the review. Figure 1 shows the study selection process.

### Quality assessment of studies

Quality assessment of the studies is presented in Additional file 2. Qualitative studies were mostly well designed. Studies using mixed methods had not explicitly explained any inconsistencies between qualitative and quantitative results and/or the risk of non-response bias in the quantitative component [33–39]. Mixed method studies were, however, included in the review as RHC is a relatively ‘young’ research field and these studies added important insights to the review while addressing at least two of the three research questions.

### Study characteristics

The methods used to study RHC varied in the studies: fifteen were qualitative [40–54], and five used mixed methods [33–36, 39]. The methods used in the studies published in books, however, were mainly qualitative [55–68] except two studies that used mixed methods [37, 38].

Studies reported in peer-reviewed journals were mostly conducted in developed countries: the United Kingdom [35, 40, 42, 44, 46, 53], the United States of America [33, 43, 51, 52], Finland [45], Australia [39, 48, 50], Denmark [48–50, 54], Norway [47] and Israel [36]. Two studies were conducted in developing countries: Brazil [41, 51] and South Africa [34]. For studies published in books, all were conducted in developed countries: the United Kingdom [58, 60, 65, 66], New Zealand [62, 67], Norway [57, 61], France [55], Switzerland [56], Australia [59], Denmark [63], Canada [64], the United States of America [68], Japan [38] and one unstated, possibly USA [37].

### Descriptions and conceptualisations of RHC

Table 1 shows descriptions of RHC, aims of the included studies, methods used to study and factors that develop RHC. Understanding RHC descriptions and forming concepts are prerequisites to moving from theory level to practical level. Table 2 summarises the underpinning RHC capabilities or categories for describing and conceptualising RHC in empirical studies.

**Table 1:** Descriptions, aims and methods used to study and factors that develop RHC

	Study reference	Description of RHC	Aim of study	Methods used to study RHC	Factors used to develop RHC
Peer-reviewed articles					
1.	Gittell, J 2008 (USA) [33]	• Organisational resilience … incorporates insights from both coping and contingency theories. It refers to the maintenance of positive adjustment and the ability to flourish or thrive amid adverse conditions when rigidity might otherwise be expected.	• Explore the role of relationships and organisational practices in enabling workers to respond in a resilient way to external pressures.	• Archival data.	• Relational coordination between professionals by sharing goals, knowledge and mutual respect.
				• Interviews.	• Frequent, timely, accurate and problem- solving communication for effective coordination.
				• Observations.	
				• Surveys.	
2.	Mash B, J, et al. 2008 (South Africa) [34]	• The organisation’s ability to remain true to its core values, competencies and vision rather than invest in a specific structure.	• Explore how to create more successful practice teams based on doctors and nurses experience.	• Interviews.	• Staff meeting and discussion with an ongoing exchange of ideas and experiences.
				• Observations and documentation of changes in progress markers and success of strategies.	
					• Communication with respect, appreciation and trust.
					• Teamwork that enables health care professionals to easily interact and commit to each other.
				• Structured questionnaire.	
					• Effective leadership by sharing the vision, and identifying values.
					• Feedback for reflection and learning.
3.	Brattheim B, et al. 2011 (Norway) [47]	• … process variation related to flexibility is an integral part of how actors deal with uncertainty, variability and high risk, enhancing safety in unpredictable settings. The resilience engineering approach to managing variations centres on attention to essential properties of adaptive behaviours.	• Identify the characteristics and sources of abdominal aortic aneurysm process variability within and between different hospitals. • Develop suggestions for how to design IT-based process support to enhance resilience in this process.	• Observations.	• Capability of awareness.
				• Semi-structured interviews.	• Capability to gain knowledge from experience.
					• Reduce unintended process variation.
4.	Nemeth C, et al. 2011 (USA) [43]	• The ability of systems to mount a robust response to unforeseen, unpredicted, and unexpected demands and to resume or even continue normal operations.	• Develop information and communication technology to support crisis management in healthcare settings.	• Observational study.	• N/A
				• Cognitive task analysis.	
				• Interviews.	
		• Resilience is an emergent property of systems that is not tied to tallies of adverse events or estimates of their probability.		• Artefact analysis.	
				• Work domain analysis.	
		• Studies how people at all levels of an organization try to anticipate paths that may lead to failure, to create and sustain strategies that are resistant to failure, and to adjust tasks and activities to maintain margins in the face of pressure to do more and to do it faster.		• Process tracing.	
				• Rapid prototyping.	
				• Evaluation.	
		• A resilient system can adjust its functioning prior to, during, or following changes and disturbances so that it can sustain required operations, even after a major mishap or in the presence of continuous stress.			
		• The notion of resilience frees safety research from hindsight bias by making it possible to understand how workers anticipate possible adverse outcomes and act in advance to avert them.			
5.	Ross A, et al. 2012 (UK) [44]	• The capacity of a system to adapt safely to changing conditions. Resilience can be defined as the ability of a system to self-correct and adapt to disturbances so that normal operations can be maintained even when unexpected conditions are encountered.	• Investigate how clinical staff deliver inpatient diabetes care.	• Interviews.	• Understanding the nature of the gap and how front-line practitioners bridge it and sometimes fail.
				• Cognitive task analysis.	
			• Identify how resilience is created and/or breaks down.		• Specialist team to coordinate decision making for various medical conditions that open a line for education, detecting problems and managing them early.
			• Provide a basis for designing interventions to improve care.		
					• Good feedback, communication and monitoring.
					• Updating knowledge.
6.	Crowe S, et al. 2014 (UK) [35]	• The capability of a health system to mitigate the impact of major external disruptions on its ability to meet the needs of the population during the disruption.	• Explore the feasibility of assessing resilience across local health services and develop a computer software to assess resilience of different service reconfigurations in the NHS in England.	• Computer software modelling tool to assess resilience.	• N/A
				• Optimisation and heuristic methods to capture response.	
7.	Clay-Williams R, et al. 2015 (Australia and Denmark) [48]	• N/A	• Investigate whether FRAM can be used to identify process elements in a draft guideline in order to develop a new guideline that aligned with WAD.	• FRAM.	• Realign WAI with WAD in implementing guidelines.
				• Meetings.	
8.	Drach-Zahavy A, et al. 2015 (Israel) [36]	• Identify, correct and ‘bounce back’ from errors, with obvious positive consequences for patient’s safety.	• Examine the relation between the strategies used during handovers and the type and number of errors in the following shift.	• Observations.	• Face to face communication between health care professionals and non-professional workers with patients.
				• Data extraction from patient’s chart.	
				• Surveys.	• Interactive discussion between incoming and outgoing health care professionals that enhances safety through situational awareness.
					• Exposure to a diversity of opinions.
9.	Sujan M, et al. 2015 (UK) [46]	• The ability of a system to adjust its functioning prior to, during, or following changes and disturbances, so that it can sustain required operations under both expected and unexpected conditions.	• Demonstrate how the study of handover’s everyday clinical work can contribute novel insights into a common and stubborn patient safety problem.	• Observations.	• Dynamic, and context-dependent trade-offs.
				• Semi-structured interviews.	• Staff experience.
				• Process mapping.	• Intuition.
					• Reconcile the gap between WAI and WAD.
					• Verbal communications.
					• Performance variability.
10.	McCray J, et al. 2016 (UK) [42]	• Team Resilience is a team’s ability to “bounce back” and “maintain” performance under adverse circumstances. Performance is the team outputs and delivery, and in the case of integrated teams in the health and social care sector, is likely to be linked to service user outcomes.	• Explore the making of resilient team from the perspective of managers in health and social care organisations.	• Focus groups.	• Effective teamwork.
					• Team relationship.
			• Identify factors that affect team performance.		
11.	Wachs P, et al. 2016 (Brazil, USA) [51]	• The intrinsic ability of a system to adjust its functioning prior to, during, or following changes and disturbances, so that it can sustain required operations under both expected and unexpected conditions. In turn, performance adjustment means filling in the gaps of standardized operating procedures, whatever their extent and reason.	• Investigating resilience skills in emergency departments by understanding how interactions between the elements forming a socio-technical system give rise to resilience skills.	• Observations.	• Individuals and Team Factors:
				• Critical decision method interviews.	➢ Collaborative work.
				• Questionnaires.	➢ Matching capacity and demand.
				• Documents analysis.	➢ Communication.
				• Meetings.	➢ Recognise the impact of small actions and decisions.
					➢ Prioritise actions and decisions.
					➢ Identify contextual factors that can hinder performance.
					➢ Anticipation of the need for actions.
					➢ Managing the trade-off between times allocated to care patients and number of patients seen.
					➢ Re-plan the sequence of activities.
					➢ Leadership.
					➢ Workarounds involving the use of equipment and materials.
					• Organisational factors:
					➢ Contingency plans for crisis management.
					➢ Standardisation of managerial and care processes.
					➢ Support for collaborative work.
					➢ Computerised system.
					➢ Management of human and material resources.
					➢ Measures to deal with lack of beds for admitted patients.
12.	Back J, et al. 2017 (UK) [40]	• The intrinsic ability of a health care system to adjust its functioning prior to, during, or following events (changes, disturbances and opportunities), and thereby sustain required operations under both expected and unexpected conditions.	• Examine escalation policies in theory and practice using RHC principles.	• CARE model.	• Team work structure.
				• Analysis of escalation policies.	• Awareness of the state of the hospital system based on experience and expertise.
				• Observations.	
				• Interviews.	
13.	Larcos G, et al. 2017 (Australia) [39]	• … refines safety by promoting flexibility rather than compliance with protocols, guides and training.	• Identify the rate and nature of interruptions the nuclear medicine technologists experience.	• Observations.	• Responsiveness by reacting effectively when a situation changes.
				• Linear regression analysis.	
			• Identify strategies that support safety in the workplace.	• Discussions.	• Attentiveness by taking appropriate action considering the situation at hand.
			• Suggest quality improvement strategies in nuclear medicine that may complement those derived from incident reporting.		• Anticipation.
					• Experience.
14.	Pickup L, et al. 2017 (UK) [53]	• Refers to how well a system is designed to recognise and respond to such shifts within an organisation and the impact on how a system function. A resilient system would be capable of identifying and adapting to potential vulnerabilities or threats to safety without the need for an incident or accident to occur.	• Understand why performance might vary in blood sampling in acute hospital settings and how a Safety-II approach can inform future safety management programmes.	• FRAM.	• N/A
				• Observations.	
				• Semi structured interviews.	
15.	Raben DC, et al. 2017 (Denmark) [49]	• … focuses on how healthcare systems succeed by rapidly responding and adapting performance in everyday work.	• Asses the feasibility of the LIIM and the challenges or difficulties revealed in the process of blood sampling.	• FRAM.	• N/A
				• LIIM.	
				• Observations.	
			• Identifying leading indicators for blood sampling among patients in a Biomedical Department.	• Semi-structured interviews.	
				• Focus groups.	
				• Walk-throughs.	
16.	Damen NL, et al. 2018 (Australia and Denmark) [50]	• N/A	• Understand and compare WAI and WAD in preoperative anticoagulation management.	• FRAM.	• N/A
				• Interviews.	
			• Examine the utility of FRAM to reconcile WAI and WAD.		
17.	Merandi J, et al. 2018 (USA) [52]	• Resilience is an essential part of Safety II. Safety II requires an “adjustment to functioning,” which goes beyond “good catches” (situations in which error is avoided by performing an expected task).	• Identify factors in a hospital system and individuals that support increased resilience in delivering patient care.	• Focus groups.	• Individuals and team factors:
					➢ Situational awareness.
					➢ Experience and expertise.
		• Resilient systems require humans to learn from what goes right and develop adaptations and flexibility to incorporate that learning going forward.			➢ Recognising the inevitability of error.
					➢ Teamwork.
					➢ Effective, open and clear communications.
					➢ Training.
					➢ Careful examination and feedback after errors.
					➢ Double- check.
					➢ Prioritising work.
					➢ Commitment to standard procedures.
					➢ Bridging experience from other microenvironments.
					• Structural and environmental factors:
					➢ Familiarity and proximity.
					➢ Shift resource availability.
18.	Raben DC, et al. 2018 (Denmark) [54]	• N/A	• Investigate how complex processes produce positive outcomes despite variability in the early detection of sepsis using FRAM.	• Document reviews.	• Experience.
				• Focus groups.	• Ability to multi-task.
				• Observations.	
				• Interviews.	
				• FRAM.	
19.	Rosso C, et al. 2018 (Brazil) [41]	• The ability of the health care system to adjust its functioning prior to, during, or following changes and disturbances, so that it can sustain required performance under both expected and unexpected conditions.	• Develop and test a framework design, which combines insights from lean production and RE.	• FRAM.	• Creation of conditions to design and construct systems that have the capacity of resilience.
				• Stream mapping.	
				• Notes from observations, focus groups and other documents.	• Modelling designs by developing innovative artefact to solve practical problems and make scientific contribution.
		• Resilience in health care … shed light on the gap between WAI and WAD, as well as on new approaches for patient safety, which rely on learning from every day work, instead of only from adverse events.			
20.	Wahlström M, et al. 2018 (Finland) [45]	• The intrinsic ability of a system to adjust its functioning prior to, during, or following changes and disturbances, so that it can sustain required operations under both expected and unexpected conditions.	• Explore surgeons’ adaptations to situational demands within robotic surgery.	• Core-task analysis.	• Mindfulness characterises: anticipation, backups, holistic consideration of patient anatomy, and thoughtful damage minimisation.
				• Action-perception-cycles.	
				• Observations.	
				• Video analyses.	• Technical developments and medical knowledge.
				• Interviews.	• Situational interpretation.
				• Self-confrontation video sessions.	
				• Workshops.	
Book chapters					
21.	Cuvelier L, et al. (France) [55]	• The intrinsic ability of a system to adjust its functioning so that it can sustain required operations under both expected and unexpected conditions.	• Identify strategies used by anaesthesiologists to avoid negative consequences of variability in everyday work.	• Open-observations. • Incidents. • Interviews.	• Care protocols. • Experience. • Making situations more predictable. • Increase knowledge. • Vocational training. • Cognitive trade-off. • Mobilisation of additional resources.
		• It is not only the system’s ability to cope with unforeseen variability that fall outside the expected areas of adaptations, but also looks at its ability to operate in foreseen conditions.			
		• A resilient system is the one capable to detect that the conditions have changed, to assure transition to another state and to operate in the new state of resilience achieved.			
22.	Pariès J, et al. (Switzerland) [56]	• The ability to make sacrificing decisions, such as accepting failures to reach an objective in the short term to ensure another long-term objective, or ‘cutting one’s losses’ by giving up initial ambitions to save what is essential.	• Observe how the ICU in the University Hospital of Geneva was functioning after the merger of two hitherto separate units.	• Observation grid.	• Anticipation capacity.
					• Skills and accuracy of team’s perception.
			• Understand how and why the merger units succeeded or failed in controlling variations.		• Trade-offs.
					• Diversity of experiences.
		• The ability to acknowledge the need to shift from one mode to the other. It measures the quality and robustness of trade-offs; their stability in the presence of disturbances.			• Interactions with patients.
					• Intuition.
					• Sacrificing decisions.
					• Functional reconfiguration.
					• Collaboration between different job profiles.
					• Strong team spirit.
					• Leadership mechanisms.
					• Flexible delegation.
23.	Laugaland K, et al. (Norway) [57]	• The ability of health care system to succeed under varying conditions to increase the proportion of intended and acceptable outcomes.	• Explore how different wards and units in hospital and primary care adjust their functions to sustain new demands imposed by system reforms.	• Observations.	• Multi-faceted outcomes from different perspectives.
				• Interviews.	• Interconnections between systems.
		• Adjustments could be deemed successful from one perspective but not from the viewpoint of others.			
		• Different outcomes thus represent different judgement of values that need to be explored and acknowledged in order to be able to share a common ground on what constitutes acceptable, successful outcomes.			
24.	Stephens RJ, et al. (USA) [68]	• Capacity for manoeuvre.	• Analyse strategies taken by staff for regulating capacity for manoeuvre in terms of RE concepts.	• Observations.	• Coordinate adaptive capacities across units.
					• Regulate the capacity for manoeuvre.
					• Reduce the risk of decompensation in hospital units.
					• Reciprocity.
25.	Anderson JE, et al. (UK) [58]	• The ability of the health care system to adjust its functioning prior to, during, or following events (changes, disturbances and opportunities), and thereby sustain required operations under both expected and unexpected conditions.	• Investigate how care of older people was delivered, how decisions were made and how people adapted to pressure in clinical environment.	• Interpretive approach.	• Balance different goals during discharge process.
				• Observations.	• Plan and co-ordinate the different tasks for discharge across different staff groups, agencies, and families and carers.
				• Interviews.	
			• Design and implement interventions to increase the safety and quality of care.	• CARE model.	
		• … ability or capacity for adaptation, rather than a state of the system.			
		• Understands the complexities of the whole system rather than focuses on a discrete part.			
26.	Debono D, et al. (Australia) [59]	• Adapt, flex and navigate competing demands so as to adjust under expected or unexpected conditions in order to sustain required operations.	• Explore nurses’ role and explanations of workarounds when using electronic medication management systems to understand the gap between WAI and WAD.	• Comparing WAI (process mapping) with WAD (observations, interviews and focus groups).	• Workarounds.
		• The shifting and jostling demands of delivering care that prioritise one goal over another in a continually changing way, the role of context in influencing that process, and ongoing judgements about when to use [or not use] primary and secondary workarounds.			
27.	Deutsch E, et al. (Unstated) [37]	• Reinforcing appropriate actions and resources making the margins and constraints of the system visible, and developing team behaviours that have the potential to improve the adaptive capacity of the team.	• Explore the role of simulation to understand and support the emergence of RHC.	• Simulation.	• N/A
				• NASA-TLX score.	
				• Debriefing.	
				• Analyse the simulation performance from the perspective of four abilities for resilience.	
28.	Furniss D, et al. (UK) [60]	• It can adjust its functioning prior to, during, or following events (changes, disturbances, and opportunities), and thereby sustain required operations under both expected and unexpected conditions.	• Investigate if the RMF can be used to extract resilience strategies during interviews.	• RMF.	• Provide an alternative means for clinicians to access relevant medical information.
				• Semi-structured interviews.	
			• Explore resilient strategies in anaesthetic’s environment.		• Take time for mental preparation.
					• Take drugs and equipment to emergency calls.
					• Maximise information extraction.
29.	Heggelund C, et al. (Norway) [61]	• N/A	• Explore the resilience mechanisms used in maternity services in two Norwegian hospitals.	• Theoretical framework using the four cornerstones of resilience: anticipation, monitoring, learning, and response.	• Identify the content and evaluate the variability in the four cornerstones of resilience.
					• Flexible organising.
				• Qualitative interviews, focus group interviews, field notes from observations (meso and micro level) and analysis of national documents (macro level).	• Cultural factors (openness, support, communication, cohesion and trust).
					• Mixing experienced and inexperienced people.
					• Knowledge and experience.
					• Buffer of staff familiar with the services.
					• Procedures and the use of checklists and protocols.
					• Simulation.
					• Multi and inter disciplinary training.
					• Teamwork.
					• Statistics available for employees.
30.	Horsley C, et al. (New Zealand) [62]	• The ability of the health care system to adjust its functioning prior to, during or following events (changes, disturbances, opportunities) and thereby sustain required operations under both expected and unexpected conditions.	• Assess aspects of team functioning in a Critical Care Complex, describe elements of a functional team and how this forms a foundation to adapt to different situations using a Team Resilience Framework.	• Team Resilience Framework.	• Shared understanding of current situation.
				• Simulation.	• Allocate or self-nominate roles to team staff.
				• Interviews and in-practice observations.	• Efficient communication.
					• Explicit about expectations.
		• The ability to adapt over multiple timescales that marks the concept of resilience as different from concepts of robustness or rebound, in which temporary stressors on the system (i.e., patient admissions, acute events, disasters) must be absorbed without overt failure.			• Know what to monitor.
					• Flexible response to events.
					• Learn why things go right.
					• Open and productive team climate.
					• Debriefings.
		• RHC should expand its aspiration beyond safety or even ‘sustaining operations’ to seeing the potential for this approach to advance health care towards the long-held goals of safe, patient-centred care delivered by engaged staff.			• Checklists.
					• Team training.
					• Human factors teaching.
					• Shared team concept.
					• Psychological safety.
31.	Hounsgaard J,et al. (Denmark) [63]	• N/A	• Elucidate the impact of variability on everyday work in a spine centre.	• FRAM.	• Mnemonic systems.
				• Interviews.	
32.	Hunte G, et al. (Canada) [64]	• The ability of a system to adjust its functioning prior to, during, or following events (changes, disturbances and opportunities), and thereby sustain required operations under both expected and unexpected conditions. Central to this proactive approach is the understanding that safety is dynamic, emerges from everyday practice, and is something a system does.	• Evaluate the RAG to develop a context-specific framework to be used by emergency care providers and ancillary staff and leaders to assess and monitor over time.	• Dialogue workshop.	• Team-environment.
				• RAG.	• Exploitation of resources.
					• Systematic (re)prioritisation.
					• Effective linkages, communication and attention to cross-scale interactions.
		• In a resilient system, large increases in work processed contribute to only small increases in recovery, and the system is able to keep pace.			
33.	Nakajima K, et al. (Japan) [38]	• To promote resilient health care, it is essential to understand how health care professionals actually work in a given environment. One way to understand everyday clinical work is based on the concepts of work-as-imagined and work-as-done.	• Understand how work is actually done for handling KCL concentrate injection solutions in Japanese hospitals.	• Direct and indirect approaches to represent WAD (minutes and memoranda of hospital committees, medication supply data, observations, interviews, and expert opinions).	• Resource allocation.
					• Systemic approach.
34.	Ross A, et al. (UK) [65]	• … to study responding, monitoring, anticipation and learning at all levels.	• Explore how delivery of care happened in inpatient diabetes care by using the CARE model to guide their interpretation.	• CARE model.	• Inpatient care cycle.
				• Interviews using cognitive task analysis techniques.	• Workarounds and outcome trade-offs.
					• Distributing expertise at the ward level.
35.	Sujan M, et al. (UK) [66]	• RHC is able to reconcile the gap between the way everyday clinical work unfolds WAD with the way managers and administrators think about clinical practice WAI.	• Evaluate how safety cases are used in health care systems.	• Process map and FMEA.	• Communication and building trust between stakeholders.
				• FRAM.	
			• Understand the gap between WAI and WAD in clinical handovers in emergency care.		• Proactive and mindful.
36.	Zhuravsky L, (New Zealand) [67]	• The ability of the health care system to adjust its functioning prior to, during, or following events (changes, disturbances and opportunities), and thereby sustain required operations under both expected and unexpected conditions.	• Demonstrate the practical application of RHC approach on sustained nursing performance after the Christchurch earthquake in New Zealand in 2011.	• Autoethnographic methodology.	• Leadership (individual and shared).
					• Simulation and debriefings.
					• Training.
					• Workarounds.
					• Proactive monitoring of signs of stress, fatigue and anxiety.
					• Utilise technical capabilities.
					• Handovers.
					• Double-loop approach to learn.
					• Realignment of WAI with WAD.

**Note:** N/A is used when studies did not report methods used to study and/or factors to develop resilience

*CARE* Concepts for Applying Resilience Engineering, *FRAM* Functional Resonance Analysis Method, *FMEA* Failure Mode Effects Analysis, *ICU* Intensive Care Unit, *IT* Information Technology, *KCL* Potassium Chloride, *LIIM* The Leading Indicators Identification Method, *NASA-TLX* The National Aeronautics and Space Administration- Task Load Index, *RAG* Resilience Analysis Grid, *RE* Resilience Engineering, *RMF* Resilience Markers Framework, *UK* The United Kingdom, *USA* The United States of America, *WAD* Work As Done, *WAI* Work As Imagined

**Table 2:** Underpinning RHC capabilities or categories used to conceptualise RHC in the included studies

RHC capabilities or categories/ study reference	[34]	[35]	[36]	[37]	[38]	[39]	[40]	[41]	[42]	[43]	[44]	[45]	[46]	[47]	[48]	[49]	[51]	[52]	[53]	[54]	[56]	[57]	[58]	[59]	[60]	[61]	[62]	[63]	[65]	[66]	[67]	[68]	[69]	Total
**Respond/cope**	**√**		**√**								**√**														**√**									**4**
**Anticipate/foresee**											**√**																							**1**
**Learn/recover**									**√**												**√**													**2**
**Monitor, respond, anticipate and learn.**					**√**		**√**	**√**				**√**	**√**		**√**									**√**			**√**	**√**						**9**
**Adjust/adapt to variability**								**√**	**√**				**√**	**√**				**√**	**√**	**√**	**√**			**√**		**√**			**√**	**√**		**√**		**13**
**Create shared vision and collective values**		**√**																																**1**
**Maintain robust response**											**√**																							**1**
**Bounce back**				**√**						**√**																								**2**
**Re-align between Work As Imagined and Work As Done**						**√**		**√**	**√**					**√**		**√**	**√**	**√**												**√**	**√**	**√**		**10**
**Trade-offs**					**√**			**√**							**√**	**√**						**√**		**√**	**√**					**√**				**8**
**Succeed**																							**√**											**1**
**Sustain system capacity for manoeuvre**																																	**√**	**1**

**Note:** Three studies [49, 54, 63] were not included as they did not include relevant information to conceptualise RHC

Although the descriptions and conceptualisation of RHC varied across studies, most shared Hollnagel’s (2017) [69] four capabilities of RHC: anticipate, monitor, respond and learn [37, 40, 44, 45, 58, 61, 62]. Other studies conceptualised RHC to be about prioritising goals in the midst of competing demands (the quality of trade-offs) [37, 40, 47, 48, 56, 58, 59, 65] or reconciling the gap between Work As Imagined (WAI) and Work As Done (WAD) [38–41, 46, 48, 65–67]. Two studies described RHC as the ability to bounce back from errors by maintaining a positive adjustment to flourish amidst adverse situations [36, 42]. There was one study that illustrated success as a cornerstone capability for RHC. The study found that successful outcomes should be interpreted from multiple perspectives (management, staff, patient, next of kin, hospital and primary care) and that the assessment of successful outcomes depends on what group perspective the focus is on [57]. Interestingly, only one study defined RHC and resilience capabilities as emergent phenomena, which arise from interactions between different variables. Such phenomena might be either desired or undesired and cannot be developed in a fully controlled way, however, it could be influenced [53].

### Methods for studying RHC

#### Data collection methods

Methods were categorised as direct or indirect sources, as described by Hollnagel et al. (2019) [12] (see Table 1).

A *direct source* is one where participants directly express their experience of how work takes place in practice. Direct sources used in included studies included interviews [33, 34, 38, 40, 43–47, 49–51, 53–59, 61–63, 65, 66]; focus groups [41, 42, 49, 52, 59, 61]; surveys and/or questionnaires [33, 34, 36]; process mapping sessions [46, 66]; and an autoethnographic approach in which the author relied on self-reflection to explore his experience while connecting this to a wider context [67].

An *indirect source* is one where participants are observed for a period or the data is collected from non-human resources. Indirect sources drawn upon in included studies were observations [34, 36, 38–41, 43–47, 49, 51, 53, 55, 57–63, 65, 68]; work domain analysis, process tracing, and artefact analysis [43]; simulation [37, 62]; patient charts [36]; document analysis (local or national guidelines, incident reports, minutes of hospital committees and medication supply data) [38, 40, 41, 48, 50, 51, 55, 56, 61]; and The National Aeronautics and Space Administration Task Load Index (NASA-TLX), a widely used multidimensional tool to assess perceived workload [37].

All studies collected data either at the micro and/or meso level (health care practitioners, managers, local guidelines). Only seven reported the use of macro-level data collected from different stakeholders, national surveys, organisation and process design documents, as well as computer software, to assess organisational resilience [35, 38, 48, 50, 51, 56, 66].

#### Tools for studying RHC

Some studies developed and/or used models, frameworks and quasi-models to study RHC. These are described here under three headings, based on RHC constructs: 1. Performance variability and WAD, 2. Cornerstone capabilities of RHC and 3. Integration with other safety management paradigms.

#### Performance variability and WAD

Ten studies developed and/or evaluated tools for studying RHC based on understanding variability in everyday clinical work, and how health care practitioners adapt and cope in response to varying conditions [40, 48–50, 53, 54, 58, 60, 63, 65]. The Concepts for Applying Resilience Engineering (CARE) model developed by Anderson et al. (2016) [70] was used in various studies to examine escalation policies used in emergency departments [40], to develop practical tools to study resilience and identify potential quality improvement initiatives [58], and to explore the misalignment between demand and the ways in which clinical staff adjust their work to be able to perform as needed [65]. The Resilience Markers Framework (RMF) was used to uncover resilience strategies used by anaesthetists, and to allow participants to reflect on their work demands and to contrast routine and non-routine aspects [60]. Different studies used the FRAM method to differentiate between WAI and WAD and to develop context-specific models in different clinical settings such as a preoperative anticoagulation management [50], blood sampling [49, 53], clinical guidelines implementation [48] early detection of sepsis [54] and a Medical Department’s daily variations and adjustments [63]. Models developed in these studies were used to elucidate the complexity of everyday clinical work, understand the variability in daily routines and suggest new perspectives to improve safety.

#### Cornerstone capabilities for resilience

While the notion of the four capabilities of RHC, anticipate, monitor, respond and learn, is not presented as a theory, Hollnagel et al. (2019) suggested using it as a generic model or quasi-model for resilience performance [12]. Five studies were based on these four capabilities [51, 56, 61, 62, 64]. The Observation Grid Model was developed to observe how an Intensive Care Unit (ICU) functioned after the merger of two hitherto separate units, and to understand how and why the merger units succeeded or failed in controlling variations [56]. A theoretical framework based on the four capabilities of resilience was used to identify what mechanisms shaped resilience in maternity services in two different hospitals [61]. The Team Resilience Framework was designed to retrospectively assess aspects of team functioning in a Critical Care Complex, describe elements of a functional team, and determine how this forms a foundation to adapt to different situations [62]. The Resilience Analysis Grid (RAG) was adapted to a context-specific framework for an urban Emergency Department in Canada and then evaluated to find discrepancies, coherence and complementarity with reference to RAG [64]. A model for describing resilience skills was proposed to identify the origin of resilience skills, contextual factors that could affect them, and leverage points that support their development in emergency departments in two different countries [51].

#### Integration with other safety management paradigms

In order to operationalise RHC, three studies highlighted the benefits from the integration between RHC and other management concepts and theories. For example, the Model of Relationships and Resilience was developed based on coping and contingency theories arguing that resilience responses require both psychological and organisational resources [33]. The framework for supporting a work system design was developed and tested in a health care system involving patient flow from an Emergency Department to an ICU. Insights from lean production (improving efficiency) and resilience engineering (improving safety) were combined in an approach to system design inspired by complexity theory [41]. When operationalising RHC, researchers will be confronted with both what goes right and what goes wrong. Sujan and colleagues (2019) combined Safety-I and Safety-II ways of thinking to ensure that stakeholders appreciated the current safety position by understanding the gap between WAI and WAD [66].

There was one tool not assigned to any category: a computer software tool to assist in decision-making concerning services’ reconfiguration in the National Health Service (NHS) in England. They used operational research techniques such as mathematical optimisation and heuristic methods to capture responses and to assess the impact of a given disruption on the capability of the health care system to respond [35]

### Factors to develop and enhance RHC

Operationalising RHC aims to find measures that reliably capture the concept under study [21]. Based on the analysis of the included studies, seven key factors were used at different levels (individual, team, and organisation) to develop RHC:

1. **Teamwork** was considered a factor in developing and sustaining resilience in the health care sector [19, 36, 40, 42, 44, 46, 51, 52, 61, 62, 64, 66, 67]. Aspects of teamwork included:

- *Effective and frequent team meetings* involving active listening, disagreement resolution and decision-making [34, 36, 42].
- *Effective communication*, characterised by respect, building trust between health care professionals, enhancing staff satisfaction to exchange information and ideas before and after the implementation of new practices [34, 36, 44, 46, 51, 52, 61, 62, 64, 66].
- *Effective leadership*, keeping the organisation focused on key objectives while also remaining open to feedback from clinical staff to create a shared vision, and revise decisions if required [19, 34, 42, 51, 66].
- *Effective involvement of clinicians* as top-down leaders to look for positive work practices [42].
- *Effective team working structure* between doctors, nurses and patient flow-coordinator roles to enhance the ability to expedite patient transfer to manage crowding in an emergency department [40, 51].

2. **In-situ practical experience** was a core factor in building resilience by providing a deep knowledge of how the system works and how the organisation adapts to and copes with expected and unexpected situations. Experienced health care professionals may teach novices how the health care system works and how to perform work. Managing different situations and cases will provide health care professionals with knowledge and experience that allows development of the resilient behaviours of anticipating, learning, monitoring and responding when facing similar situations [39, 40, 45–47, 56, 61, 65]. Another example of building resilience is in-situ simulation training and debriefings, which provide opportunities for experts and novices to understand practice and adapt to routine and unexpected situations [44, 61, 62, 67].
3. **Exposure to diverse views and perspectives on the patient’s situation** provided the fundamental advantage of understanding the patient’s situation thoroughly while decreasing the likelihood of cognitive bias and maintaining the previous level of performance. One example was face-to-face verbal communication in handovers, with interactive questioning and a summary written by the outgoing nurse helping to decrease the probability of bias that might occur through inappropriate assumptions of an incoming nurse [36, 52, 56, 57, 61, 62].
4. **Trade-offs:** The clinical staff dynamically used their subjective assessment of the current situation to resolve stressors and tension. Being mindful and acting proactively to shift from one mode to another in the presence of disturbances is one of the key reported factors in developing RHC [39, 46, 51, 55, 56, 58, 64–67].
5. **The value of using protocols and checklists:** Protocols and checklists are valuable ways of defining potential variabilities and situations that are well known in the clinical practice and well described in the literature of speciality. [52, 53, 56, 63].
6. **System design:** One empirical study developed and tested a framework in a health care environment by adopting insights from resilience engineering to create conditions that supported resilient performance. Eight design propositions were developed which can contribute to the redesign of socio-technical systems to be safe and efficient at the same time [41].
7. **Workarounds:** These facilitated practice to continue by enabling staff to cope with challenges and maintain effective delivery of patient care [59, 65, 67]. One study considered that intended workarounds were necessary activities to mitigate risk and enhance safety [47].

## Discussion

The aim of this systematic review was to identify and understand how RHC is conceptualised and operationalised including methods used to study and factors identified to develop RHC. Most studies conceptualise RHC based on Hollnagel’s (2017) [69] four capabilities of RHC: anticipate, monitor, respond and learn. Methods for studying RHC include the use of a variety of data sources (direct and indirect, and mainly qualitative) and existing or new tools/frameworks. Factors that develop and enhance RHC include effective team relationships, trade-offs and health care ‘resilience’ training of health care professionals.

### Conceptualisation of RHC

Concept formation is essential to inform and guide operationalisation efforts. Recent studies have conceptualized RHC by understanding the gap between WAI and WAD, which shifts the focus to everyday clinical work instead of adverse events only, and the importance of reconciling this gap to enhance RHC [38, 40, 41, 46, 48, 50, 65–67]. Perhaps unsurprisingly for a research area that has only developed in the last half a decade, most studies assessed shared the same definition for RHC, i.e. that developed by Hollnagel and colleagues. We share the perspective of Ellis et al. (2019) that RHC would benefit from more research of an international nature, to overcome this conformity of ideas and over-reliance on the founding authors [18]. Although the four cornerstones of RHC capabilities defined by Hollnagel have contributed to a deeper understanding of the RHC concept, and provided insights into how to operationalise in health care systems, the four capabilities affect and are affected by the environment [69]. Consistent with other systematic reviews [11, 17], this review found that other capabilities such as flexibility, trade-offs, and robustness should be taken into consideration to conceptualise RHC.

### Methods for studying RHC

A health care system is viewed as a complex adaptive system comprising networks of components (hospitals, health care professionals, families and patients) that interact in non-linear and evolving ways [71]. All included studies indicated that researchers acknowledged the complexity in health care settings – they triangulated data from different data sources (documents, reports, interviews and observation). Few studies, however, have used methodological triangulation (quantitative and qualitative) to study RHC.

Most of the studies included in this systematic review used qualitative data to study everyday clinical work that explained frontline practitioners’ contribution to RHC and kept patients safe despite pressure. Although interviews and focus groups are widely used in qualitative research, the assumption that participants’ words are indicators of their inner experiences may be questionable [72]. Observational research reports what people do or say, rather than what they say they do. Observation, however, can include a high degree of researcher bias as the method relies on interpretation of what has been observed. The researcher cannot ‘see’ attitudes and memories, so it can be difficult to create an accurate analysis from observation alone. One study used self-confrontation video sessions in which participating surgeons were encouraged to explain what took place while they were conducting surgery [45]. To understand in-situ practices, describe the complexity of health care, and model and test specific kinds of recommendation to improve safety and resilience, more innovative approaches should be used to explore WAD in-situ from the perspective of frontline practitioners. Video reflexive ethnography (VRE), developed by Iedema and colleagues [73], could be used to explore WAD. Video footage of real-time practices is shown back to participants in small groups where they collectively reflect to make sense of their work and negotiate meaningful, context-appropriate ways of improving their practices [73–76].

While the extensive use of qualitative methods is one of the strengths of RHC, deepening understanding of everyday clinical work rather than merely measuring system behaviour [18], other methods have been under-explored. Quantitative methods such as surveys, mathematical methods and computer software modelling tools could have fruitful implications. Mixed method approaches could be used to determine the outcomes of applying RHC principles and to investigate the extent to which RHC principles have been used at the various organisational levels (staff, patient, team, managers and organisation) [11, 18].

Several of the studies included developed models/frameworks based on predefined resilient constructs. The performance variability and WAD construct brings resilience closer to an empirical ground by facilitating understanding of how everyday situations and uncertainties are successfully managed [12]. This construct has been shown to be relevant in identifying and assessing ways in which performance variability can be monitored and managed, e.g. in the Vessel Traffic Service system [77] and in the Air Traffic Management System which has been used to analyse a mid-air collision over the Amazon [78]. Various studies included in this systematic review used FRAM to develop models that were essential in understanding system functions, performance and variability [48–50, 53, 54, 63]. Consistent with the literature [79], this systematic review found that it is imperative to combine FRAM models with quantitative data to quantify functions and measure outputs of functions in order to assess distributions of variability.

Patriarca et al. (2018) found that the RAG model, which comprises questions related to the four cornerstone capabilities has not been used widely [17]. However, the findings of the current systematic review showed that four studies used the generic principles of RAG as the basis on which more context-specific grids or questions were developed and tailored [56, 61, 62, 64]. The Resilience Engineering Tool to Improve Patient Safety (RETIPS) tool developed by Hegde et al. (2019) used RAG as an initial framework to guide the development of a more specialised tool to build the resilience profile of a system. The tool was validated and revised based on feedback from clinicians, resulting in a version customised for anaesthesia residents. RETIPS has been developed to operationalise the Safety-II paradigm by learning how things go well in everyday clinical work [80].

Despite the importance of the complementary perspective to learn from incidents and to understand how everyday clinical work is successful, few studies have used studied RHC from both perspectives [55, 56, 66]. In order to operationalise RHC, researchers need to bridge between RHC and other safety paradigms to enhance patient and organisational safety. Our recommendation is consistent with other studies that advocate combining data from the RHC perspective with others such as accident analysis, risk assessments, grand rounds, and electronic health report data to enhance system safety through the identification of visible outcomes, unnoticed deficiencies and longitudinal implications of certain adaptations [81, 82]. Some models used to study RHC had not incorporated sufficient details to enable problems to be understood and/or resolved in meaningful and comprehensive ways [35]. Current methods for studying RHC represent efforts to improve the understanding of RHC. Health care settings are constantly experiencing turnover of staff, policies and equipment, so future studies will need to investigate the practicality and feasibility of the methods, enhance their applicability and evaluate interventions for generalisation across organisations.

### Factors to develop and enhance RHC

Recognising that the health care environment is complex and unpredictable, whilst also understanding how the system works in everyday and unexpected situations, is a starting point for improving patient safety. Few studies have taken a whole-system approach to developing resilience in health care. These results reflect those of Berg et al. (2018), who also found that multi-level mechanisms for studying RHC are not well established [22]. Almost all the included studies did not assess how the factors used by individuals and teams affect the resilience of the whole system. The identified factors that were used to enhance safety could make sense locally, but the outcomes are not necessarily successful at the higher levels. Indeed, resilient performance and adaptations could lead to negative outcomes at the organisational level [17, 57]. Laugaland et al. [57] found that adjustments could be deemed successful from one perspective (hospital) but poor from the viewpoint of others (patients). This review supports evidence from previous reviews [18, 21, 22] to suggest that the focus should be on how resilience is distributed throughout the entire system at different levels, in different settings, cultures and countries, to help better understanding of RHC. The move from research to practice is still nascent. More work is needed to design interventions based on the identified factors and then to measure their effectiveness in different health care contexts, investigate the implementation of designs and artefacts, and explore how to operationalise the changes.

### Future work

Several questions remain to be answered before a gold standard for studying and developing RHC can confidently be identified. We propose the following conceptualisation of RHC for consideration to underpin future research - the ability of the whole system (individual, team and organisation) to manage the gap between WAD (what goes wrong and what goes right) and WAI proactively and in response to situations while achieving patient and system safety goals. The focus of future studies should consider the following:

- Explore whether the adaptations and adjustments used are appropriate to maintain usual work, and how resilient adaptations on a system level could affect resilience on other system levels. Explore WAD by using the Integrative Learning approach [82], exploring what goes right and what goes wrong, factors that contributed to success or failure, challenges that threatened patient safety or hindered successful intervention, etc.
- Explore WAD and WAI for each system level e.g. micro, meso and macro, and integrate findings to form a robust understanding of the work system. Insights into WAD depends on the angle and system level the research has focused on and may not always reflect the everyday work of health care practitioners.
- Explore the variability in everyday work in more depth using mixed method approaches. For example, using methods that enhance reflexivity such as VRE to reflect on invisible aspects of work and also applying quantitative methods for measuring work outcomes.
- Develop and use tools and/or frameworks including integrating those from other safety paradigms capable of describing factors and mechanisms occurring at different system levels that enhance or hinder resilience.
- Explore RHC in multi-level, diverse settings (long-term care facilities such as nursing homes, outpatient clinics, ambulatory care, home health care and emergency medical services) and in different countries to build on current knowledge and guide the operationalisation of RHC to different settings and cultures.

## Review limitations

First, relevant data might be missed from unpublished studies. To counteract this limitation, a broad search was conducted to include both peer-reviewed studies published in journals and relevant studies published in books. Second, most of the studies included were conducted in developed countries and more studies are needed to investigate whether the findings are applicable to other countries. Third, resilient factors reported were derived from specific case scenarios and this might affect their influence in different settings. Fourth, mixed method studies were included in the review despite the quality of the studies as they added insights to the review. Lastly, the results of this systematic review represented the researchers’ interpretation and other researchers might have different perspectives and reach different conclusions.

## Conclusion

Most studies shared similar characteristics in their descriptions and conceptualisations of RHC. Although methods to study and factors that develop RHC currently exist, it is vital to understand how RHC works within existing health care systems, how to enhance RHC and how it can be sustained. In addition, it important to understand and explore how to develop RHC effectively in order to devise innovative interventions and to evaluate and design resilient socio-technical systems. Future research is needed to address the wider safety implications of RHC amidst organisational and institutional change.

## Supplementary information

**Additional file 1: **Mixed Methods Appraisal Tool **(**MMAT) checklist items [20].

**Additional file 2:** Quality assessment of included studies.

## Data Availability

The data used in the study are available from the corresponding author on request.

## Abbreviations

CARE: Concepts for Applying Resilience Engineering
FRAM: Functional Resonance Analysis Method
ICU: Intensive Care Unit
MMAT: Mixed Methods Appraisal Tool
NASA-TLX: National Aeronautics and Space Administration-Task Load Index
NHS: National Health Service
PRISMA: Preferred Reporting Items for Systematic Reviews and Meta-Analysis
RAG: Resilience Analysis Grid
RE: Resilience Engineering
RETIPS: Resilience Engineering Tool to Improve Patient Safety
RMF: Resilience Markers Framework
RHC: Resilient Health Care
VRE: Video Reflexive Ethnography
WAD: Work As Done
WAI: Work As Imagined
