# Supplementary material for: Resilient Health Care: a systematic review of conceptualisations, study methods and factors that develop resilience
Source: BMC Health Serv Res. 2020 Apr 17;20:324. doi: 10.1186/s12913-020-05208-3 (PMC7165381; doi:10.1186/s12913-020-05208-3)
Supplement: Supplementary file 1 — Additional file 1: Mixed Methods Appraisal Tool (MMAT) checklist items [20]. [file 12913_2020_5208_MOESM1_ESM.docx]

**Additional File 1**

Mixed Methods Appraisal Tool **(**MMAT) checklist items [[18](#_ENREF_18)].

|  | **Methodological quality criteria** |
| --- | --- |
| **1. Screening questions (for all types)** | **1.1.** Are there clear research questions?  **1.2** Do the collected data allow to address the research questions? |
| **2. Qualitative studies** | **2.1.** Is the qualitative approach appropriate to answer the research question?  **2.2.** Are the qualitative data collection methods adequate to address the research question?  **2.3.** Are the findings adequately derived from the data?  **2.4.** Is the interpretation of results sufficiently substantiated by data?  **2.5.** Is there coherence between qualitative data sources, collection, analysis and interpretation? |
| **3. Quantitative descriptive studies** | **3.1.** Is the sampling strategy relevant to address the research question?  **3.2.** Is the sample representative of the target population?  **3.3.** Are the measurements appropriate?  **3.4.** Is the risk of nonresponse bias low?  **3.5.** Is the statistical analysis appropriate to answer the research question? |
| **4. Mixed method studies** | **4.1.** Is there an adequate rationale for using a mixed methods design to address the research question?  **4.2.** Are the different components of the study effectively integrated to answer the research question?  **4.3.** Are the results adequately brought together into overall interpretations?  **4.4.** Are divergences and inconsistencies between quantitative and qualitative results adequately addressed?  **4.5.** Do the different components of the study adhere to the quality criteria of each tradition of the methods involved? |
