# Supplementary material for: Resilient Health Care: a systematic review of conceptualisations, study methods and factors that develop resilience
Source: BMC Health Serv Res. 2020 Apr 17;20:324. doi: 10.1186/s12913-020-05208-3 (PMC7165381; doi:10.1186/s12913-020-05208-3)
Supplement: Supplementary file 2 — Additional file 2: Quality assessment of included studies. [file 12913_2020_5208_MOESM2_ESM.docx]

**Additional File 2**

**Quality assessment of included studies**

| **Study reference** | **Screening questions** | | | **Qualitative**  **studies** | | | | | **Quantitative**  **descriptive Studies** | | | | | | **Mixed**  **methods studies** | | | | |
| --- | --- | --- | --- | --- | --- | --- | --- | --- | --- | --- | --- | --- | --- | --- | --- | --- | --- | --- | --- |
|  | **1.1** | **1.2** | | **2.1** | **2.2** | **2.3** | **2.4** | **2.5** | **3.1** | | **3.2** | **3.3** | **3.4** | **3.5** | **4.1** | **4.2** | **4.3** | **4.4** | **4.5** |
| Studies published in journals | | | | | | | | | | | | | | | | | | | |
| 1. Gittell J. (2008)[34] | √ | √ | | √ | √ | √ | Can’t tell | √ | | √ | √ | √ | Can’t tell | √ | √ | √ | √ | Can’t tell | √ |
| 1. Mash BJ, et al. (2008)[35] | √ | √ | | √ | √ | √ | √ | √ | | √ | √ | √ | Can’t tell | √ | √ | √ | √ | Can’t tell | √ |
| 1. Brattheim B, et al. (2011) [48] | √ | √ | | √ | √ | √ | √ | √ | | N/A | N/A | N/A | N/A | N/A | N/A | N/A | N/A | N/A | N/A |
| 1. Nemeth C, et al. (2011)[44] | √ | √ | | √ | √ | √ | √ | √ | | N/A | N/A | N/A | N/A | N/A | N/A | N/A | N/A | N/A | N/A |
| 1. Ross A, et al. (2012)[45] | √ | √ | | √ | √ | √ | √ | √ | | N/A | N/A | N/A | N/A | N/A | N/A | N/A | N/A | N/A | N/A |
| 1. Crowe S, et al. (2014)[36] | √ | √ | | N/A | N/A | N/A | N/A | √ | | √ | √ | √ | √ | √ | √ | √ | √ | Can’t tell | √ |
| 1. Clay-Williams R, et al. (2015)[49] | √ | √ | | √ | √ | √ | √ | √ | | N/A | N/A | N/A | N/A | N/A | N/A | N/A | N/A | N/A | N/A |
| 1. Drach-Zahavy A, et al. (2015)[37] | √ | √ | | √ | √ | Can’t tell | √ | √ | | √ | √ | √ | Can’t tell | √ | √ | √ | √ | Can’t tell | √ |
| 1. Sujan M, et al. (2015)[47] | √ | √ | | √ | √ | √ | √ | √ | | N/A | N/A | N/A | N/A | N/A | N/A | N/A | N/A | N/A | N/A |
| 1. McCray J, et al. (2016)[43] | √ | √ | √ | | √ | √ | √ | √ | | N/A | N/A | N/A | N/A | N/A | N/A | N/A | N/A | N/A | N/A |
| 1. Wachs P, et al. (2016)[52] | √ | √ | √ | | √ | √ | √ | √ | | N/A | N/A | N/A | N/A | N/A | N/A | N/A | N/A | N/A | N/A |
| 1. Back J, et al. (2017)[41] | √ | √ | √ | | √ | √ | √ | √ | | N/A | N/A | N/A | N/A | N/A | N/A | N/A | N/A | N/A | N/A |
| 1. Larcos G, et al. (2017) [40] | √ | √ | √ | | √ | √ | √ | √ | | √ | √ | √ | Can’t tell | √ | √ | √ | √ | √ | √ |
| 1. Pickup L, et al. (2017) [54] | √ | √ | √ | | √ | √ | √ | √ | | N/A | N/A | N/A | N/A | N/A | N/A | N/A | N/A | N/A | N/A |
| 1. Raben DC, et al. (2017) [50] | √ | √ | √ | | √ | √ | √ | √ | | N/A | N/A | N/A | N/A | N/A | N/A | N/A | N/A | N/A | N/A |
| 1. Damen NL, et al. (2018) [51] | √ | √ | √ | | √ | √ | √ | √ | | N/A | N/A | N/A | N/A | N/A | N/A | N/A | N/A | N/A | N/A |
| 1. Merandi J, et al. (2018) [53] | √ | √ | √ | | √ | √ | √ | √ | | N/A | N/A | N/A | N/A | N/A | N/A | N/A | N/A | N/A | N/A |
| 1. Raben DC, et al. (2018) [55] | √ | √ | √ | | √ | √ | √ | √ | | N/A | N/A | N/A | N/A | N/A | N/A | N/A | N/A | N/A | N/A |
| 1. Rosso C, et al. (2018)[42] | √ | √ | √ | | √ | √ | √ | √ | | N/A | N/A | N/A | N/A | N/A | N/A | N/A | N/A | N/A | N/A |
| 1. Wahlströma M, et al. (2018)[46] | √ | √ | √ | | √ | √ | √ | √ | | N/A | N/A | N/A | N/A | N/A | N/A | N/A | N/A | N/A | N/A |
| Studies published in books | | | | | | | | | | | | | | | | | | | |
| 1. Cuvelier L, et al.[56] | √ | √ | √ | | √ | √ | √ | √ | | N/A | N/A | N/A | N/A | N/A | N/A | N/A | N/A | N/A | N/A |
| 1. Pariès J, et al.[57] | √ | √ | √ | | √ | √ | √ | √ | | N/A | N/A | N/A | N/A | N/A | N/A | N/A | N/A | N/A | N/A |
| 1. Laugaland K, et al.[58] | √ | √ | √ | | √ | Can’t tell | √ | √ | | N/A | N/A | N/A | N/A | N/A | N/A | N/A | N/A | N/A | N/A |
| 1. Stephens RJ, et al.[69] | √ | √ | √ | | √ | √ | √ | √ | | N/A | N/A | N/A | N/A | N/A | N/A | N/A | N/A | N/A | N/A |
| 1. Anderson JE, et al.[59] | √ | √ | √ | | √ | √ | √ | √ | | N/A | N/A | N/A | N/A | N/A | N/A | N/A | N/A | N/A | N/A |
| 1. Debono D, et al.[60] | √ | √ | √ | | √ | √ | √ | √ | | N/A | N/A | N/A | N/A | N/A | N/A | N/A | N/A | N/A | N/A |
| 1. Deutsch E, et al.[38] | √ | √ | √ | | √ | √ | √ | √ | | √ | √ | √ | X | √ | √ | √ | √ | Can’t tell | √ |
| 1. Furniss D,et al.[59] | √ | √ | √ | | √ | √ | √ | √ | | N/A | N/A | N/A | N/A | N/A | N/A | N/A | N/A | N/A | N/A |
| 1. Heggelund C, et al.[62] | √ | √ | √ | | √ | √ | √ | √ | | N/A | N/A | N/A | N/A | N/A | N/A | N/A | N/A | N/A | N/A |
| 1. Horsley C, et al.[[63](#_ENREF_41)] | √ | √ | √ | | √ | √ | √ | √ | | N/A | N/A | N/A | N/A | N/A | N/A | N/A | N/A | N/A | N/A |
| 1. Hounsgaard J, et al.[[64](#_ENREF_42)] | √ | √ | √ | | √ | √ | √ | √ | | N/A | N/A | N/A | N/A | N/A | N/A | N/A | N/A | N/A | N/A |
| 1. Hunte G, et al.[[65](#_ENREF_43)] | √ | √ | √ | | √ | √ | √ | √ | | N/A | N/A | N/A | N/A | N/A | N/A | N/A | N/A | N/A | N/A |
| 1. Nakajima K, et al.[39] | √ | √ | √ | | √ | √ | √ | √ | | √ | √ | √ | √ | √ | √ | √ | √ | Can’t tell | √ |
| 1. Ross A, et al.[66] | √ | √ | √ | | √ | √ | √ | √ | | N/A | N/A | N/A | N/A | N/A | N/A | N/A | N/A | N/A | N/A |
| 1. Sujan M, et al.[67] | √ | √ | √ | | √ | √ | √ | √ | | N/A | N/A | N/A | N/A | N/A | N/A | N/A | N/A | N/A | N/A |
| 1. Zhuravsky L.[68] | √ | √ | √ | | √ | √ | √ | √ | | N/A | N/A | N/A | N/A | N/A | N/A | N/A | N/A | N/A | N/A |
